# Biopolymers for Enhanced Health Benefits

**DOI:** 10.3390/ijms242216251

**Published:** 2023-11-13

**Authors:** Leonard-Ionut Atanase

**Affiliations:** 1 Faculty of Medical Dentistry, “Apollonia” University of Iasi, Pacurari Street, No. 11, 700511 Iasi, Romania; leonard.atanase@yahoo.com; 2 Academy of Romanian Scientists, 050045 Bucharest, Romania

The definition of the term biopolymer is often controversial, and there is no clear distinction between “biopolymers”, “bioplastics”, and “bio-based polymers”. Biopolymers (or bioplastics) are considered by some authors to only be polymers that are biodegradable. In practice, they bring together biosourced polymers that are produced from renewable resources, as well as biodegradable polymers and even sometimes biocompatible polymers. Thus, they can be classified according to two distinct criteria: the origin of the resource from which they are produced and their end-of-life management (biodegradability).

The current biopolymers can be classified into three main groups, depending on the two aforementioned criteria: (i) biodegradable polymers from renewable resources (polysaccharides, polymers of bacterial origin, and biosynthetic polymers); (ii) biodegradable polymers from fossil resources obtained via industrial synthesis processes; and (iii) non-biodegradable polymers from renewable resources.

Biopolymers have a diverse chemical structure, and their physicochemical properties make them suitable for clinical, biomedical, and pharmaceutical applications due to their versatile characteristics, such as biocompatibility, biodegradability, and low immunogenicity, which are key features in the new approach to the design of novel advanced materials.

In this Special Issue, entitled “Biopolymers for Enhanced Health Benefits”, a total contribution of eight papers—seven original articles and one review—were published, focusing on the synthesis and characterization of different types of biopolymers for biomedical applications.

Rodriquez-Cendal et al. [1] reviewed the recent advances and potential biomedical applications of a poly(3-hydroxybutyrate-co-3-hydroxyvalerate) (PHBV) copolymer, with special emphasis on drug encapsulation and scaffold construction. It appears that this biopolymer improves drug stability and bioavailability, prolongs the drug release rate, and enhances mechanical properties, biocompatibility, and cellular interactions, which are important advantages in the field of biomedicine.

Ponjavic et al. [2] also obtained PHBV-based biomaterials as films by combining the copolymer with a strong anticancer bacterial pigment, prodigiosin (PG). The incorporation of PG increased the crystallinity, thermal stability, and morphology of the composites. Moreover, these films have a potent anticancer activity, which was demonstrated against colon cancer cells (HCT116).

Ozturk et al. [3] investigated the micellization of two different poly(ε-caprolactone)-graft-poly(N-vinylcaprolactam-co-N-vinylpyrrolidone) [PCL-g-P(NVCL-co-NVP)] graft copolymers in the presence of Dorzolamide and Indomethacin as model drugs for the potential treatment of glaucoma. A slight increase in the micellar sizes was observed in the Dorzolamide-loaded micelles, whereas the Indomethacin-loaded micelles had smaller sizes. As expected, in vitro biological tests showed that these drug delivery systems are haemo- and cytocompatible.

Gan et al. [4] investigated the preparation of a composite hydrogel, cross-linked with hydrogen bonds, based on Salecan (Sal) and soy protein isolate (SPI). It was demonstrated that, by increasing the Sal concentration, the internal network structure of the composite hydrogel was denser and more uniform. Moreover, the Sal concentration also increased the thermal stability of the hydrogel.

Dzhuzha et al. [5] studied the synthesis and self-assembly of amphiphilic polypeptides obtained via the post-polymerization modification of poly(α,L-glutamic acid) with various hydrophobic and basic L-amino acids as well as D-glucosamine. These carriers were further used for the encapsulation of Paclitaxel as a model drug and demonstrated a high cytostatic efficacy against human lung adenocarcinoma (A549).

Nurzynska et al. [6] investigated whether curdlan-based biomaterials, obtained through the incorporation of hop bioactive compounds (crude extracts or xanthohumol), can be a suitable candidate for the treatment of skin wounds. It was demonstrated by the authors that these biomaterials are non-cytotoxic and allow for the proliferation of skin fibroblasts. On the contrary, they inhibit the production of pro-inflammatory interleukin-6 by human macrophages which is important for the skin regeneration process (study on a *Danio rerio* larvae model).

Ulagesan et al. [7] prepared a hydrogel based on k-carrageenan and phycobiliprotein that was also intended for the treatment of wound healing. First of all, its antioxidant and antimicrobial activity was assessed. Furthermore, it was demonstrated that these hydrogels induce a rapid and complete wound closure after 24 and 48 h.

Masetto et al. [8] obtained magnetic-core nanoparticles functionalized by polyclonal procalcitonin antibodies (PCT) for the standardized and reproducible quantification of sepsis in human serum or HeLa cell extracts.

The papers published in this Special Issues clearly prove that the field of biomaterials is important for high-value-added applications in the medical field. However, a great deal of effort is necessary in order to translate the results obtained in this academic research to an industrial scale as well as to clinical randomized trials.
